# Germ Cell Tumor Located in Gastrointestinal System: A Report of Two Cases

**DOI:** 10.4021/wjon493w

**Published:** 2012-07-05

**Authors:** Mehmet Kucukoner, Ali Inal, Mehmet Ali Kaplan, Zuhat Urakci, Ugur Firat, Feyzullah Ucmak, Abdurrahman Isikdogan, Guven Tekbas

**Affiliations:** a Medical Oncology Department, Dicle University, Diyarbakir, Turkey; b Pathology Department, Dicle University, Diyarbakir, Turkey; c Gastroenterology Department, Dicle University, Diyarbakir, Turkey; d Radiology Department, Medicine Faculty, Dicle University, Diyarbakir, Turkey

**Keywords:** Germ cell tumor, Gastrointestinal involvement, Diagnosis

## Abstract

Germ cell tumors (GCTs) occur generally in the testes or ovaries. Extra-gonadal location of GCTs is very rare .Extra-gonadal GCTs usually arise from midline structures, the commonest sites being the retroperitoneal and mediastinum. Gastrointestinal germ cell tumors are very uncommon. The etiology, prognosis and clinical course of gastrointestinal GCTs are not well understood yet. Weherein present two GCTs cases who referred with gastrointestinal bleeding and obstruction these tumors originated from retroperitoneal site. In the light of the literature, the clinical and pathological findings of the cases are presented. Prominent features of our cases were being located in the gastrointestinal system, being at young ages, presenting with gastrointestinal bleeding and good outcome.

## Introduction

Germ cell tumors (GCTs) arise from malignant transformation of premordial germ cells. Germ cell tumors can be seminomas or nonseminomatous germ cell tumors. Nonseminomatous germ cell tumors consist of embryonal cell carcinomas, choriocarcinomas, yolk sac tumors, or teratomas. Germ cell tumors occur predominantly in the gonads (90-95%). Extra-gonadal GCTs represent 1% - 5% of all germ cell tumors [1]. Extra-gonadal GCTs usually arise from midline structures, the commonest sites being the retroperitoneum, mediastinum, sacrococcygeal region, pineal gland [2, 3]. In the cases in the literature, germ cell cancers originated from gastrointestinal system (GIS) are rarely presented in the stomach, esophagus, rectum colon and liver [4-6]. In gastrointestinal system, germ cell tumors can develop as primary or secondary to metastasis from retroperitoneal site.

The true etiopathogenesis of germ cell tumor in the GIS is as yet undetermined. According to a theory, the origin of germ cell neoplasm’s is considered to be primordial germ cells that may have been arrested along the migratory route from the hindgut yolk sac region into the embryonic genital ridge; this would account for many extragonadal germ cell neoplasm’s arising in the midline [1].

Germ cell tumors rarely metastasize to the GIS with an incidence that is less than 5%. The most frequent mode of metastasis to the GIS is direct extension from the retroperitoneal lymph nodes, which drain the testes. Ileal and jejunal metastases are more common due to their retroperitoneal locations as well as the fact that the testes have retroperitoneal lymphatic drainage. Among the GIS metastasis, the duodenum is the most uncommon location. The most common manifestations of GIS metastasis are intestinal obstructions and/or gastrointestinal bleeding [7]. Generally, gastrointestinal system adenocarcinomas are shown in old patients but GIS germ cell tumors frequently occur in the young patients. The prognosis of gastrointestinal cancer with germ cell elements is very poor because many of these patients have widespread metastases at the time of diagnosis. Therefore, aggressive combination chemotherapy can be considered in addition surgery [8].

We report two cases with primary germ cell tumors in the duodenum and colon together with their clinical and pathological characteristics.

## Case Report

### Case 1

**Figure 1:**
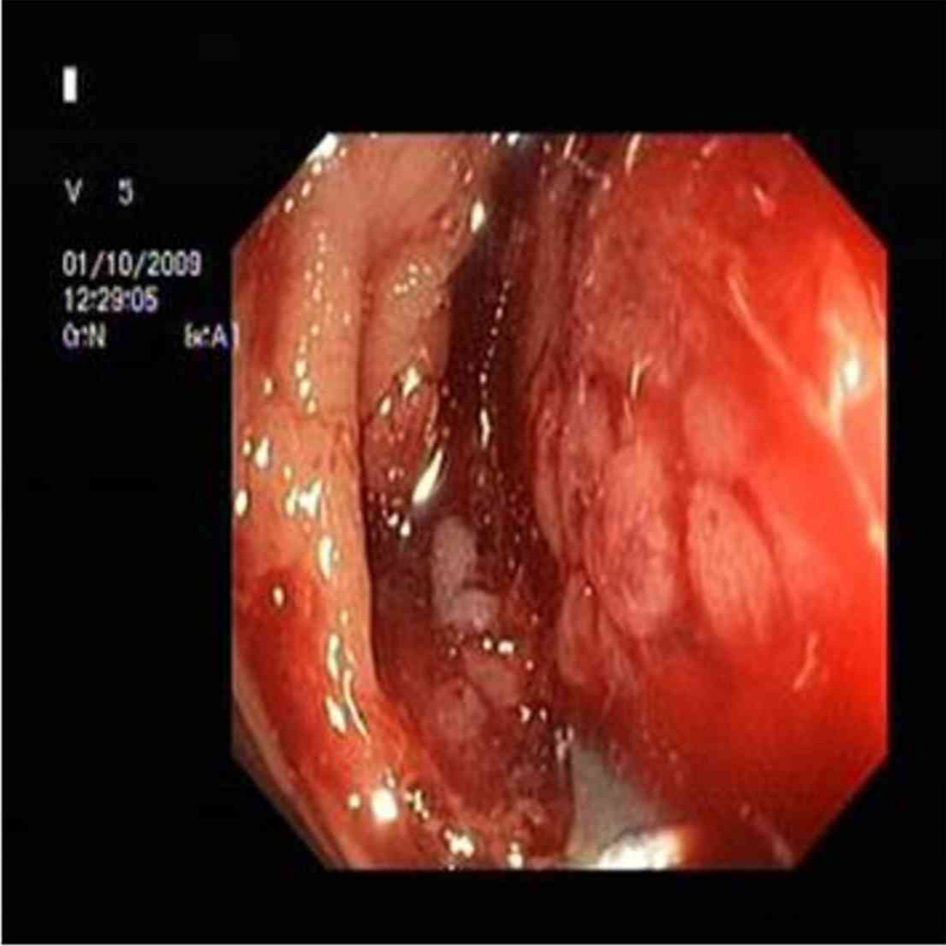
A mass in proximal colon.

A 32-year-old male patient was referred to our gastroenterology clinics with complaints of melena, abdominal pain, constipation and weight loss. Physical examination was gave no abnormality. Initial laboratory studies revealed normal results, except for hemoglobin level as 9 g/dL and hematocrit 26%. Abdominal ultrasound and tomography revealed intestinal wall thickening (about 3.5 cm) presented throughout approximately 10 cm segment of bowel, at the right abdominal region. Colonoscopy showed a mass surrounding intestinal lumen and preventing transition of colonoscopic device to proximal near hepatic flexura of colon (Fig. 1). Despite colonoscopic biopsy was applied two times, tissue samples gave non-specific histopathological appearances because of probably taken a biopsy of necrotic areas. Open laparatomy operation was performed to the patient. In the operation, frozen section samples examination revealed that the tumor was malignant, therefore hemicolectomy, lymph node dissection and ileocolic anastomosis were performed. Postoperative macroscopic examination revealed a mass 8 x 7 x 7.5 cm in size, with tumoral formations which containing large necrosis areas that invading from intestinal mucosa to serosa. Postoperative staging was T3N1M0 (Stage III).

**Figure 2:**
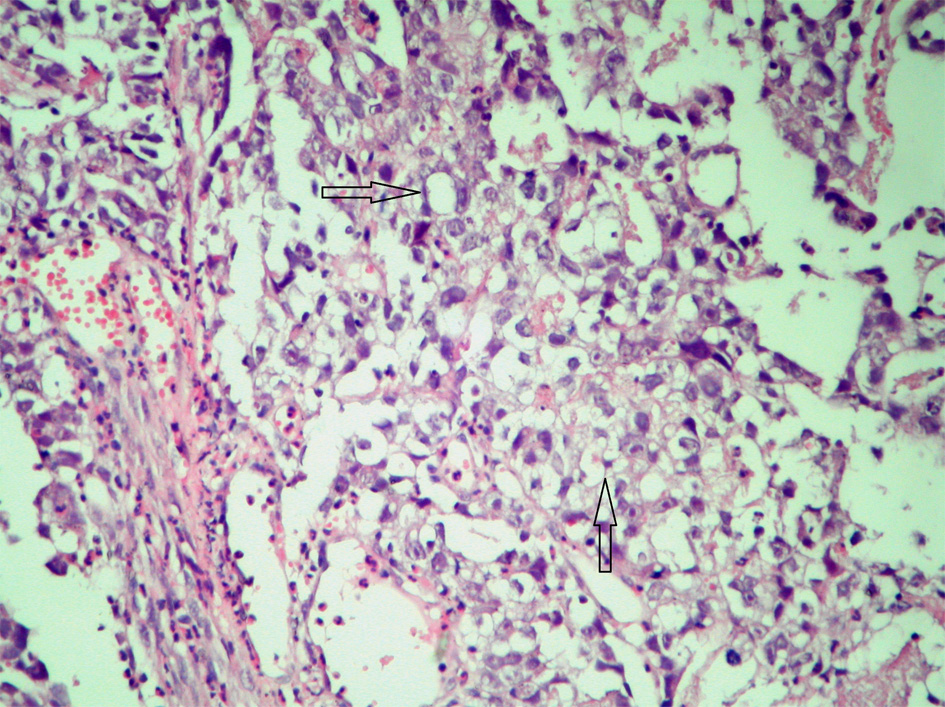
AFP reactivity in tumor cells.

Microscopically the tumor cells were found as uniform with abundant clear cytoplasm, large necrosis areas and rosette structures and with sharply outlined cell membranes containing a large centrally located nucleus with hyper chromatic feature. In many areas, the tumor cells were arranged in nests outlined by fibrous bands and immunohistochemically these cells exhibited reactivity for α-fetoprotein (AFP) (Fig. 2, Immunoperoxidase, x 400). Based on the result of histopathological examination, germ cell tumor was diagnosed especially seminoma was considered. Tumor markers of the patient, were with in the normal limits (AFP: 1.77 ng/mL, human chorionic gonadotropin (HCG): 0.1 mIU/mL LDH: 178 U/L). Ultrasonography and computed tomography imaging of gonads (testes) did not show a primary tumor the patient was accepted as clinical stage-I. Adjuvant four cycles BEP (Bleomycin, Etoposit, Cisplatin) chemotherapy was given. After completion of four cycles chemotherapy, the patient followed up in remission for 8 months.

### Case 2

**Figure 3:**
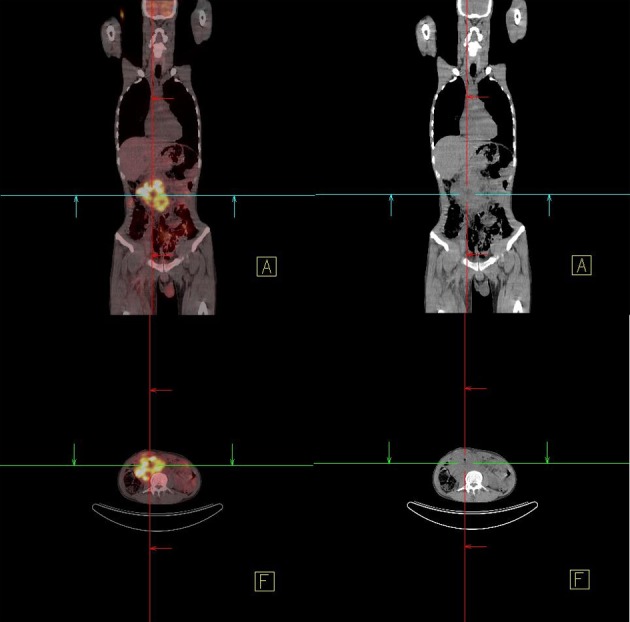
In PET-CT, a retroperitoneal mass.

**Figure 4:**
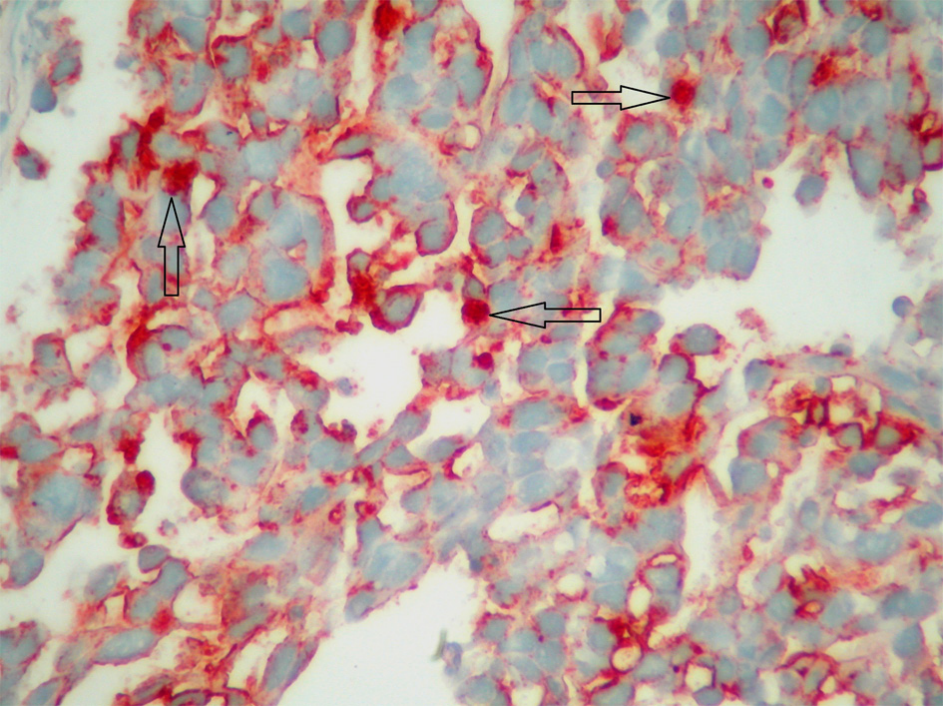
PLAP recativity.

A 34-year-old male patient was admitted with complaints of upper-gastrointestinal bleeding and vomiting. Physical examination was normal except for an epigastric palpable mass. Complete blood count gave hemoglobin 8 gr/dL and hemarocrit 25%, and LDH was 426 U/L. In endoscopy, a polypoid mass which obstructing 80% of the lumen was seen in the second part of duodenum. In computer tomography, a mass 10 x 9 x 7 cm in size was detected in duodenum. In PET-CT, a tumor from retroperitoneal site invading to duodenum was showed (Fig. 3). There was no distant metastasis. Because of upper-gastrointestinal bleeding and duodenal obstruction, palliative antecolic gastroenterostomy was performed. Since the tumor was locally-advanced stage, partial resection was applied. On microscopic examination, generally solid and some glandular architectures including zones of eosinophilic coagulative necrosis were observed in the tumor with large polygonal shaped cells showing ill-defined cytoplasmic borders and cytoplasmic vacuolation similar to lymphoblast or signet ring cells, on the other hand Immunohistochemical staining showed patchy placental alkaline phosphatase (PLAP) reactivity (Fig. 4) and focal weak alpha-fetoprotein (AFP) positivity in the tumor cells reminiscent of an embryonal carcinoma with a yolk sac tumor component. In germ cell tumors, the positive of PLAP and AFP are specific. In the result of post-operative pathology, germ cell tumor, especially embryonal cell carcinoma type was reported. Tumor markers of the patient were as follows; increased AFP (321 IU/mL), and normal HCG (< 1 mIU/mL). After palliative operation, 6 cycles BEP (Bleomycin, Etoposit and Cisplatin) were given as primary chemotherapy. Following the chemotherapy, high AFP level decreased and tumoral mass was shown to be disappeared by (PET-CT) in the follow-up control. After completion of his treatment, the patient followed -up in remission for 6 months.

## Discussion

Primary gastrointestinal germ cell tumors have been very rarely reported in the literature. Imaging studies of thorax, abdomen and pelvis of our cases revealed the tumor localized in the duodenum and colon. Moreover, duodenum and ascending colon are almost entirely retroperitoneal. Therefore, we accepted our cases as GIS metastasis of retroperitoneal germ cell tumors.

Extra-gonadal GCTs are more frequently seen in young patients with different histological subtypes and locations. Generally, primary germ cell tumors have well prognosis, whereas Extra-gonadal GCTs have a poor prognosis. Stage, site of origin, histologic type and high tumor burden are the most important prognostic factors. In the literature, the prognosis of germ cell tumors with GIS metastasis has been reported as poor. The treatment requires a multimodality approach. The treatment of localized disease is surgery, the treatment of disseminated disease is combined chemoradiotherapy [9].

Histomorphological appearance of our colon case was consisted with seminom a histological findings and our duodenal case had histological characteristics of embryonic carcinoma.

In the patients with GIS germ cell tumors, symptoms occur based on the localization of the tumor. Clinical presentation of both of our cases was gastrointestinal bleeding. In present cases, we reported germ cell tumors localized in colon and duodenum and growing intraluminally. Right colon tumors are often grown in polypoid, exophytic style. They cause occult blood loss and rarely cause obstruction. Small intestine tumors are generally determined with bleeding, obstruction, epigastric pain.

Some biochemical markers are important in the diagnosis and treatment of germ cell tumors, including HCG, AFP and lactate dehydrogenase (LDH). AFP is never elevated in seminomas, and while HCG is occasionally elevated in seminomas, levels tend to be lower than those seen in nonseminomas. LDH may be elevated in either type of tumor. Teratomas do not secrete HCG or AFP [4]. In our case with germ cell tumor in colon, serum AFP and HCG were in normal limits but LDH was in upper-high limit. However in our case with gastric germ cell tumor, serum AFP was high.

Because of our cases were not in metastatic stage, we firstly applied surgery and secondly combined chemotherapy treatments were applied to our patients. Both cases were in the remission throughout follow-up period.

Germ cell tumors should be kept in mind in the differential diagnosis of young male patients which have malignant evidence presenting with GIS bleeding. The treatment of GIS germ cell tumors should be multidisciplinary approach including surgery, chemotherapy and radiotherapy.
